# Structural Properties and Interaction Partners of Familial ALS-Associated SOD1 Mutants

**DOI:** 10.3389/fneur.2019.00527

**Published:** 2019-05-21

**Authors:** Jisen Huai, Zhongjian Zhang

**Affiliations:** Institute of Psychiatry and Neuroscience, Xinxiang Medical University, Xinxiang, China

**Keywords:** ALS, SOD1, gain-of-function, conformational disease, motor neuron death

## Abstract

Amyotrophic lateral sclerosis (ALS) is the most common motor neuron degenerative disease in adults and has also been proven to be a type of conformational disease associated with protein misfolding and dysfunction. To date, more than 150 distinct genes have been found to be associated with ALS, among which Superoxide Dismutase 1 *(SOD1)* is the first and the most extensively studied gene. It has been well-established that SOD1 mutants-mediated toxicity is caused by a gain-of-function rather than the loss of the detoxifying activity of SOD1. Compared with the clear autosomal dominant inheritance of SOD1 mutants in ALS, the potential toxic mechanisms of SOD1 mutants in motor neurons remain incompletely understood. A large body of evidence has shown that SOD1 mutants may adopt a complex profile of conformations and interact with a wide range of client proteins. Here, in this review, we summarize the fundamental conformational properties and the gained interaction partners of the soluble forms of the SOD1 mutants which have been published in the past decades. Our goal is to find clues to the possible internal links between structural and functional anomalies of SOD1 mutants, as well as the relationships between their exposed epitopes and interaction partners, in order to help reveal and determine potential diagnostic and therapeutic targets.

## Background

Amyotrophic lateral sclerosis (ALS), also referred to as Lou Gehrig's disease, is an adult-onset relentless neurodegenerative disease. It starts with a progressive paralysis and usually leads to death within 3–5 years of diagnosis due to the selective killing of the upper and lower motor neurons responsible for controlling voluntary muscle movement of speech, swallowing, respiration, and limbs (1, 2). ALS presents as a familial or a sporadic form, depending on whether or not there is a family history of the disease (3). While familial ALS (fALS), representing about 10% of all ALS cases, is due to genetic mutations and is inherited from a family member, the cause of sporadic ALS (sALS, accounting for about 90% of ALS cases) is not well-understood and is probably due to a combination of environmental and genetic risk factors (4–6). In addition, sALS and fALS cases not only present clinically indistinguishable symptoms, but also share some molecular signatures (7, 8). Currently, more than 20 genes have been associated with fALS, of which four account for the majority of familial cases which include: the Chromosome 9 Open Reading Frame 72 gene (*C9ORF72*, 40%), Superoxide Dismutase 1 (*SOD1*, 20%), Fused in Sarcoma (*FUS*, 1–5%), and TAR DNA Binding Protein (*TARDBP*, 1–5%) (4, 9). Until now, the known ALS genes explained about 70% of fALS and around 15% of sALS cases. However, despite having a relatively clear genetic background, the underlying pathogenic mechanisms still remain elusive and there is currently no effective therapy available for this disease (10, 11).

Among the known genes underlining ALS, *SOD1* gene still remains a major cause of fALS and has been very extensively investigated. This gene encodes for the detoxifying copper/zinc binding SOD1 enzyme, which has been found to be localized mainly in the cytosol, as well as in the nucleus, peroxisomes, and mitochondria (12). The first description of the ALS disease dates back to at least 1,824 by Charles Bell (13), however, *SOD1* as the first risk gene of ALS was discovered in 1993 (14, 15). Before that, scientists had already come a long way to discover that the genetic form of ALS was dominantly inherited and linked to 21q22.11 where the *SOD1* gene is located (16, 17). After 1993, research on ALS entered a new era, especially when the first *SOD1* transgenic mouse model (SOD1 G93A) was established in 1994 (18). This transgenic mouse line and its derivative lines mimicking ALS cases developed adult-onset neurodegeneration of the spinal motor neurons and a progressive motor deficit leading to paralysis and premature death by 5–6 months of age (18, 19). Currently, over 180 different mutations throughout the five exons of the *SOD1* gene have been described, the majority of which are missense point mutations resulting in a dominant mode of inheritance causing more than 160 disease-associated variations spread over the entire 154 amino acid sequence (20, 21).

The SOD1 enzyme is highly conserved throughout evolution and makes up 1–2% of the total soluble protein content in the central nervous system (22, 23). In addition, the expression of SOD1 is ubiquitous and neither restricted to nor increased in the spinal cord and motor neurons, and SOD1 levels are not developmentally regulated (12). At present, it is still unclear how SOD1 mutations selectively cause motor neuron death. It has been reported that the presence or absence of endogenous wild-type SOD1 has no obvious effect on mutant-induced ALS progression (24). Moreover, neither haploinsufficiency nor a dominant negative mutation has been proven to be the underlying mechanisms of SOD1 mutant associated ALS, because all of the originally identified and most recently found mutants are missense mutations without loss-of-function and knocking out of *SOD1* is not able to recapitulate disease phenotype either (14, 15, 21, 25). All these lines of evidence indicate that SOD1 mutants cause disease most probably via a gain-of-function, although it has also been proposed that a loss-of-function might play a modifying role in ALS (26–28). A great number of cellular mechanisms have been suggested to be potentially involved in the pathogenesis of SOD1-fALS; however, distinguishing cause from effect and identifying the critical processes remains challenging (29). Here, in this review, we will focus on the structural variations and properties of SOD1 mutants and their corresponding interaction partners, which are tightly related to widening the array of gained functions of the SOD1 mutants which cause selective motor neuron depletion. This may shed new light on the pathogenesis and therapeutic strategies of SOD1-fALS.

## Structural Properties of fALS-Associated SOD1 Mutants

**Figure 1:**
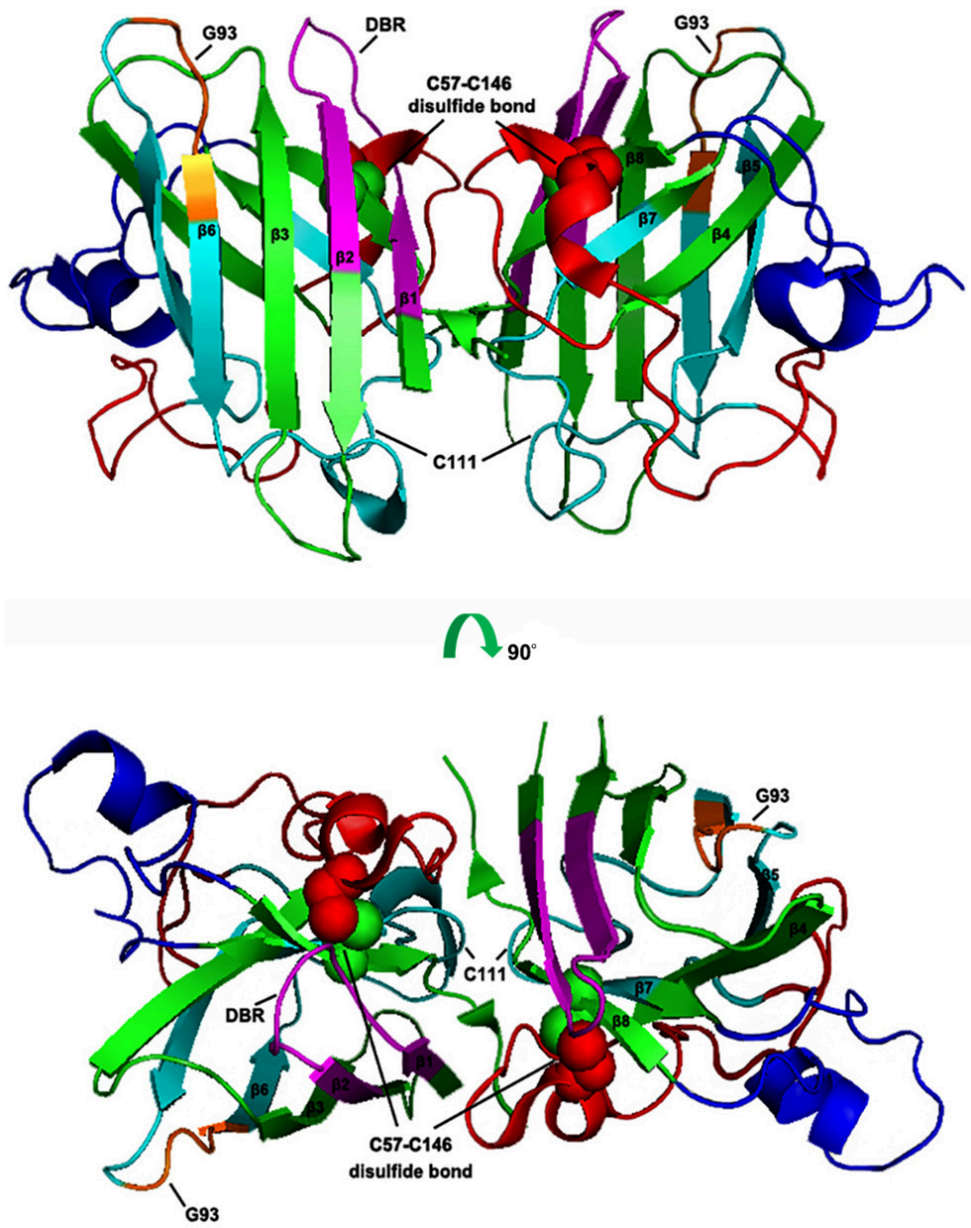
Schematic presentation of human SOD1 tertiary structure. The X-ray crystallographic structure of human wtSOD1 (PDB 2C9V) modeled in PyMOL, including the eight ß sheets (ß1: 2–8 aa; ß2: 15–22 aa; ß3: 29–36 aa; ß4: 41–48 aa; ß5: 83–89 aa; ß6: 95–101 aa; ß7; 116–120 aa; ß8: 143–151 aa), seven loops and one α helix (134–137 aa), DBR segment (5–18 aa, in Magentas), C4F6 epitope (80–118 aa in Cyans, in which 92–96 aa are core components, in Oranges), the disulfide bond (the balls in Green and Red).

SOD1 is a 16 kDa protein and normally forms a 32 kDa homodimer. The architectural structure of each SOD1 subunit consists of a β-barrel core and seven loops at the edge which are held together by an intramolecular disulfide bond, a binuclear metal binding site and a global hydrogen bond network. The metal binding site holds a copper and a zinc ion and is responsible for the catalyzing activity of SOD1 (30–32) (Figure 1).

### Both Cu/Zn Metalation and Intrasubunit Disulfide Bonds Are Critical for Maintaining the Structural Integrity of SOD1

Usually, the eukaryotic wild-type SOD1 is remarkably stable, and is able to maintain an intrasubunit disulfide bond even in the reducing environment of the cytosol and is active under a variety of stringent denaturing conditions (33). Previous studies have shown that the unusually stable quaternary structure (Homodimer) of human SOD1 is controlled by both its metal occupancy and its C57-C146 disulfide status (33–36), and loss of the Cu/Zn cofactors and disruption of the disulfide bonds may result in pathogenic misfolding (37). It has been reported that the status of the intrasubunit disulfide bonds and the metalation of the molecule are able to mutually influence each other. In particular, breaking of the disulfide linkage can cause metal loss (34, 38), indicating that the posttranslational intrasubunit disulfide bond formation plays a critical role in the maintenance of SOD1 in its functional holo state (along with the metal ions) (39). This is corroborated by further experimental results showing that removal of the intrasubunit disulfide bonds can set free the interface loop IV and make it more flexible, thus leading to loss in metal binding (40).

Clearly, the protein's disulfide bond formation controls the Cu/Zn coordination (41). Similarly, the correct coordination of Cu/Zn to SOD1 is also critical for the formation of disulfide bonds and the maintenance of its structural integrity (35, 42). Recently, it was found that Cu coordination induces the disulfide bond formation of SOD1 via the copper chaperone for superoxide dismutase (Ccs1) (43), whereas the Zn coordination drives the local folding of two disordered loops into a catalytic subdomain and stabilizes the structure of SOD1 (44).

### The Common Conformational Alterations of ALS-Associated SOD1 Mutants

Mutant SOD1 can adopt multiple misfolded conformations, and different structural variants mediate different aspects of fALS (45). Thus, the characterization of common conformational alterations of ALS-associated SOD1 mutants should be particularly challenging. It has been claimed that SOD1 can present more than 44 states depending on metal occupancy, disulfide status and oligomeric state (46), and many site mutations in SOD1 can induce structural and functional defects resulting in ALS (15). Until now, more than 180 point mutations throughout all the five exons in human SOD1 have been reported, and more than 160 mutants have been found to be associated with ALS (20, 47). Although the properties of pathogenic SOD1 mutants are considerably different in terms of protein stability, catalytic activity, and metal binding, a common pattern has been observed. That is, most ALS-associated mutations have the greatest effect on the immature form of SOD1, destabilizing the metal-free and disulfide-reduced polypeptide to the point that it becomes unfolded at physiological temperatures (Tm<37°C) (46).

It was recently shown that all pathogenic SOD1 mutants except mutations in the Derlin-1 binding region (DBR) have a common property, namely, an association with Derlin-1, a component of the endoplasmic reticulum-associated degradation machinery (48, 49). The reported Derlin-1 binding region (5-18AA at the N terminal of SOD1) has been found to be a common toxic segment of almost all ALS-related SOD1 mutants. This epitope, when exposed in malformed SOD1 mutants can be detected by the antibody MS785 produced against the SOD1 mutant (48, 49). MS785 is the first antibody to be able to distinguish ALS-linked toxic SOD1 mutants from both wild-type and nontoxic mutants, and it was suggested to be used as an innovative tool for the diagnosis of ALS. It was shown to recognize endogenous SOD1 in B lymphocytes derived from 14 ALS patients carrying SOD1 mutations but not from 11 healthy controls (49). Since the MS785 reactive epitope is located at the dimer interface, its exposure indicates that the dimer is destabilized/reoriented or even monomerized, otherwise, the epitope should be concealed in the dimeric junction interface (30, 37, 48). Until now, whether the Derlin-1 binding conformation adopted by the pathogenic mutants is an immature monomer or a dimer or another form *in vivo* has not been proven. However, according to the registered structures of the human SOD1 mutants at the Research Collaboratory for Structural Bioinformatics (RCSB) protein data bank (PDB), most of the analyzed site mutants including those with mutations at the dimer interface can still populate the dimeric structure akin to wild-type SOD1 (Table 1). Conversely, the truncated forms of SOD1 with the removal of loops IV and VII which result in total metal loss, or with the removal of the Cu/Zn ligands which causes Cu or Zn loss, present only as monomers (Table 1). It is noteworthy that almost all the site mutants, including the SOD CallA (40) (Table 1) which abolishes disulfide bond formation, and H46R/H48Q (50, 51) (Table 1) which demonstrates compromised Zn binding, can still present as dimers in a crystal, indicating that most of the ALS-associated mutants except the truncated ones such as SOD1 L126Z (52) (Table 1) can also form dimers *in vivo*, but these malformed SOD1 mutants should be unstable due to a dysfunctional disulfide bond and abnormal metal coordination (40). The adoption of a dimeric form by these site mutants in a crystal might be explained by the fact that the local changes induced by the site mutations are insufficient to prevent the occurrence of dimerization. However, they may cause a retardation of the dimerization and/or changes in the global structure of the molecule which will lead to a reorientation or shift of the dimer interface (37) and/or ß sheets (30), resulting in an exposure of toxic epitopes such as DBR, etc. This is consistent with the report that even a single hydrogen bond strain relative to the correct geometry for Cu binding can cause global structural motions (53). Nevertheless, it is necessary to keep in mind that the structures of the same molecule in solution and in a crystal may differ, since conditions such as the temperature for crystallization can be controlled, while they cannot be controlled *in vivo*. It has been reported that the SOD1 protein in solution is highly disordered, with a large range of conformations and that the apostate crystallizes only at low temperatures, suggesting that crystallization is most stable in solution (54). Thus, ALS-associated site mutants *in vivo* may present similar dimeric structures as in a crystal, which should be more stable than other soluble structures including monomers, trimers, and other forms. However, these dimeric conformations usually represent rearranged forms occurring due to site mutations and are themselves unstable and prone to interact with other molecules and form aggregates (30, 31, 39).

**Table 1:** Overview of registered structures of WT and mutant human SOD1 (summarized from protein data bank (https://www.rcsb.org) up to now).

	**Monomer**	**Dimer**	**Octamer**	**Decamer**
WT		2C9V, 3T5W, 5O3Y, 5O40, 4FF9, 3RE0, 3KH3, 3KH4, 1PU0, 1L3N, 5U9M, 2AF2		
WT apo form	6FLH, 1RK7, 1KMG			
Single site mutant	2MP3, 2NAM	5K02, 1UXM, 3GZQ, 1AZV, 2WYZ, 2WZ0, 2WYT, 2WZ5, 1PTZ, 1OEZ, 1OZT, 4OH2, 1OZU, 4MCM, 4MCN, 3H2Q, 3QQD, 3CQP, 3CQQ, 2VR6, 2VR7, 2VR8, 2ZKW, 2ZKX, 2ZKY, 3GZO, 2WZ6, 2WKO, 3GZP, 1UXL, 4A7T, 4A7U, 4A7V, 4A7S, 3ECV, 4A7Q, 4A7G, 3H2P, 1P1V		
Double site mutant		1PU0, 2GBT, 3GQF, 2NNX, 3K91, 3ECW, 3ECU		
Mutant with more than two site mutations	1MFM	2R27, 2GBU, 2GBV		
Mutant with Loops IV and VII depletion	5J07, 5J0C, 5J0F, 5J0G, 2XJK, 4BCZ			
Mutant with Loops IV and VII depletion plus site mutation	4XCR, 4BD4			
Mutant with other fragment depletion	3HFF, 1KMG, 2XJL			
SOD1 residues 28–38			6B79, 5DLI, 5IIW	
SOD1 residues 30–35			5WMJ	
SOD1 residue 101–107				4NIN
SOD1 residue 147–153 with or without site mutation				4NIP, 4NIO

### The Diverse Toxic Properties of Mutant SOD1

The maturation process of SOD1 involves many steps and is very complex (38, 55, 56). The maturation process of SOD1 takes place in the cytosol. Pathogenic mutations are proposed to retard the post-translational maturation, decrease the structural stability, and hence trigger the misfolding of SOD1 proteins (56). Especially, the immature fraction on ribosomes is suggested to produce species favoring misfolding and aggregation over folding to the native dimeric state (38). Therefore, it might also be true that different mutants are misfolded in the same conformation the DBR is expressed, but the global structure is not the same, since DBR is only a small segment of the molecule (48). Nevertheless, DBR may function as the lowest common denominator (57). For example, there are two different sets of mutations, one set (H46R, G85R, D124V, D125H, and S134N) leads to impaired metal binding with disordered electrostatic and zinc-binding loops, whereas another set (A4V, L38V, G41S, D90A, and G93A), although with normal zinc ion incorporation, is more sensitive to both a disulfide reducing agent and a metal chelator compared with wild-type SOD1, yet both sets exhibit a similar aberrant hydrophobic behavior, suggesting that different mutations may lead to the same structural property via different processes (34).

Recently, non-native trimeric SOD1 has been reported to be associated with neurotoxicity (58). The aggregated fibril form, which was previously believed to be toxic and to cause motor neuron death, displayed even protective roles in neuron-like cells. Interestingly, a newly discovered C4F6 epitope within the cytotoxic SOD1 species was found to be expressed on the surface of the trimer (59, 60). The C4F6 epitope shows a soluble pathogenic conformation that is common to misfolded SOD1 variants, the key residues of which include Asp(92) and Asp(96) (60, 61). Although the C4F6 antibody recognizes a conformation-dependent epitope common to many SOD1 mutants associated with ALS pathology, the C4F6 reactive forms are not detected by all antibodies that recognize other misfolded SOD1 species (62). There are several notable differences between the epitopes recognized by C4F6, SEDI (SOD1 Exposed Dimer Interface), and USOD (Unfolded SOD1) antibodies. C4F6 is reactive for a conformational epitope including G93, which is distal to the epitopes recognized by SEDI and USED (62). Moreover, it was reported that oxidized wild-type SOD1 in the motor neurons of the lumbosacral spinal cord of a subset of human sALS cases share the same C4F6 epitope with SOD1 mutants (62). Interestingly in another report, C4F6 was shown to react only with SOD1 mutants in ALS affected tissues and cells, whereas the tissues and cells not affected by the disease containing high levels of mutant proteins were not recognized by C4F6 (63), indicating that in ALS affected tissues or cells, the SOD1 mutant can adopt the forms recognized by C4F6. Until now whether the epitopes recognized by MS785 and C4F6 are present in parallel on the same conformation or whether they just represent different pathogenic conformations is unclear and worthy of extensive investigation. The epitopes most likely arise simultaneously or sequentially at different stages of SOD1 aggregation (64) because exposure of both C4F6 and MS785 epitopes is modulated by mutations affecting the SOD1 electrostatic (loop VII) and zinc binding (loop IV) loops (49, 60).

Furthermore, multiple misfolded SOD1 species may exist, for example, demetalated mutants have an increased affinity for mitochondria and are readily detected by the misfolded SOD1 antibodies B8H10 and DSE2-3H1, while misfolded SOD1 accumulated on mitochondria can also be labeled with another set of conformational specific antibodies such as AMF7-63 (45, 65). It has been proposed that improper protein maturation and incompletely folded states render the two aggregation-prone segments from the C terminus, (101)DSVISLS(107) and (147)GVIGIAQ(153), available for interaction (66). Covalent bis-ANS labeling of spinal cord extracts revealed that alterations in the surface hydrophobicity of the H46R/H48Q mutations in SOD1 provoked the formation of high molecular weight SOD1 species with lowered solubility, likely due to an increased exposure of the hydrophobic surfaces (67). Additionally, the large conformational variability of the apostate allows the free reduced cysteine Cys-6 to become highly accessible in solution, whereas it is essentially buried in the metalated state and in the crystal structures. Such solvent accessibility, together with that of Cys-111, accounts for the free cysteine-dependent oligomerization and/or aggregation tendency of the apostate of SOD1 (54, 68). Besides, the SOD1 residues 28–38 can assemble into a corkscrew structure in ALS-associated SOD1 mutants (69). Therefore, identifying the relationship between these specific epitopes/sites and the changes in the interaction partners due to the exposure of new epitopes/sites on the molecular surface will be of great significance for establishing an effective therapeutic strategy against ALS.

## Interaction Partners of Mutant SOD1 and Their Toxic Properties

**Figure 2:**
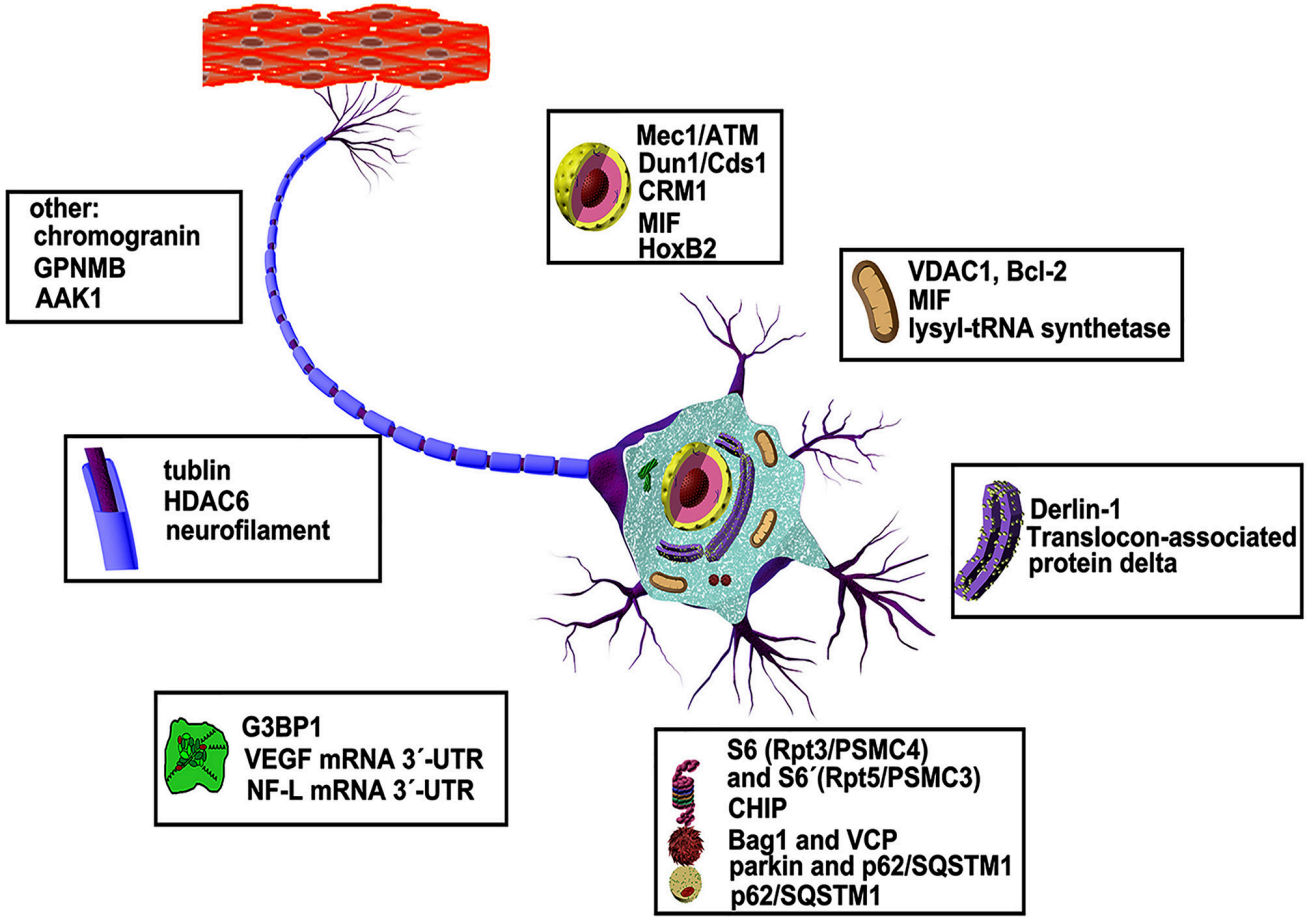
Abnormal interaction partners of mutant SOD1 and underlying mechanisms. More than dozens of molecules in various organelles of motor neurons have been shown to be defective in animal models and patients of ALS disease (see text for details).

ALS-associated mutations of SOD1 can cause a global variation in structure which may result in a gain of toxicity, yet the underlying mechanism is incompletely understood. In particular, it is not clear which parts of the molecule contain poisonous stings and how they work. Investigation using anti-SOD1 antibodies has yielded new structural insight into SOD1 misfolding as mentioned above (70). However, how the soluble forms of these mutants survive degradation before forming aggregates and to which molecules they bind are not fully understood. Identification of new partners that can selectively interact with the malformed SOD1 mutants and investigation of their potential roles in ALS is important for discovering new pathways involved in disease pathology. Here, we will summarize the proteins and other molecules that have been found to date to bind specifically to SOD1 mutants (Figure 2), in anticipation of finding some common rules based on the basic functions of these specific interaction partners.

### SOD1 Mutant Interaction Partners Related to Gene Transcription

Besides its canonical function as an antioxidant enzyme, wild-type SOD1 has also been shown to play an important role as a nuclear transcription factor (71). Normally, wild-type SOD1 is present mainly in the cytoplasm and nuclei of motor neurons, in response to general ROS (instead of its substrate superoxide) the cytoplasmic SOD1 rapidly relocates into the nucleus. This process has been shown to be mediated by Mec1/ATM and its effector Dun1/Cds1 kinase, through Dun1 interaction with SOD1 and regulation of SOD1 by phosphorylation at S60 and S99. In the nucleus, phosphorylated SOD1 binds the promoters and regulates the expression of oxidative resistance and repair genes (72, 73). However, many SOD1 mutants related to fALS have been found to be removed from the nucleus and are distributed mainly in the cytoplasm, suggesting that the toxicity of mutant SOD1 might originate from either a loss of its transcription function or its protection of the nucleus from the active enzyme (27, 74). Furthermore, the abnormal clearance of SOD1 mutants from the nucleus was found to be carried out by chromosomal maintenance 1 [CRM1, also known as exportin 1 (XPO1)], which can specifically bind to many misfolded SOD1 mutants harboring exposed nuclear export signal (NES)-like sequences on their surfaces. The consensus NES-like sequence is normally buried inside the molecule in the wild-type state in the nucleus. When expressed on the molecular surface, it can bind with CRM1 and with anti-NLP and other two antibodies D3H5 (epitope residues: 24–55) and USOD (epitope residues: 42–48) produced for recognizing misfolded SOD1 (74).

Recently, it was reported that macrophage migration inhibitory factor (MIF), a multifunctional protein with cytokine/chemokine activity and cytosolic chaperone-like properties, inhibits the nuclear clearance of SOD1 mutants when overexpressed in motor neuron-like NSC-34 cells. Moreover, MIF reduces the toxicity of misfolded SOD1 by directly interacting with it. The chaperone function and protective effect of MIF in neuronal cultures do not require intrinsic catalytic activities (75). However, the above-mentioned data about the clearance of SOD1 mutants from the nucleus is challenged by another report indicating that SOD1 mutants (G93A and G37R) prominently accumulate in the nuclei of spinal motor neurons of transgenic mice (76). This discrepancy in results is difficult to interpret and may result from differences in experimental conditions such as the cell types and animals used. In another report using SOD1 G93A transgenic mice, an SOD1 mutant was found co-localizing with homeobox B2 (HoxB2), a homeodomain-containing transcription factor usually localized in the nuclei of motor neurons and redistributed together with the SOD1 mutant to perikaryal and proximal neurites of motor neurons (77). Thus, generally speaking, it is more convincing that SOD1 mutant tends to be removed from the nucleus into the cytoplasm instead of accumulating in the nucleus. In *Caenorhabditis elegans* it was suggested that SOD1 mutants are removed from the nucleus by CRM1 as a defense mechanism against the proteotoxicity of misfolded SOD1 in the nucleus (74). Whether the clearance of SOD1 mutants from the nuclei of transgenic mouse motor neurons is through interaction with CRM1 or not, and can the SOD1 mutant still play transcription factor roles in this scenario are not clear. Further careful investigations are warranted. In addition to being a multifaceted cytokine, MIF is involved in a variety of neurological diseases (78). Therefore, it will be particularly important to study whether SOD1 mutants cause ALS via interaction with native MIF, leading to its dysfunction or depletion.

### SOD1 Mutant Interaction Partners Related to Mitochondrial Dysfunction

It has been reported that misfolded SOD1 accumulates on the cytoplasmic faces of mitochondria by binding directly to a voltage-dependent anion channel (VDAC1) and/or Bcl-2 (79, 80). VDAC1 is an integral membrane protein embedded in the outer mitochondrial membrane and forms channels that control the flux of ions and metabolites across the mitochondrion, thus mediating the organelle's cross-talk with the rest of the cell. It interacts with both pro-apoptotic and anti-apoptotic factors and is therefore a gatekeeper for mitochondria-mediated cell death and survival signaling pathways (81). Direct binding of SOD1 mutants to VDAC1 inhibits its conductance and this reduction in VDAC1 activity has been shown to diminish the survival of SOD1 mutant mice (79). BCL-2, another protein localized to the outer membrane of mitochondria, plays an important role in promoting cell survival and inhibiting the actions of pro-apoptotic proteins. In cells, mice and patient spinal cords, when bound with an SOD1 mutant, Bcl-2 is converted into a toxic protein due to a conformational change which exposes its toxic BH3 domain (80). Interestingly, this toxicity requires simultaneous mitochondrial localization of an SOD1 mutant and Bcl-2 as well as also Bcl-2 related proteins such as Bax and Bak. Truncation of the Bcl-2 transmembrane domain to release Bcl-2 in the cytoplasm or deletion of Bax/Bak abolishes damage of the outer mitochondrial membrane and cytochrome c release, impairs cell viability, and delays disease onset in SOD1^G93A^ mice (80, 82). Moreover, the overexpression of MIF also significantly reduces the association of misfolded SOD1 with both mitochondria and endoplasmic reticulum (ER) membrane and rescues from mutant SOD1-induced cell death (83). These results indicate that both the interactions between the misfolded SOD1 mutant with VDAC1 and Bcl-2 are organelle specific and may have a synergistic effect on the induction of mitochondrial dysfunction and cell death.

Except for VDAC1 and Bcl-2, the mitochondrial form of lysyl-tRNA synthetase (KARS) is the first described member of mitochondrial proteins whose interaction with SOD1 mutant contributes to mitochondrial dysfunction in ALS (84, 85). Aminoacyl-tRNA synthetases (ARSs) are enzymes responsible for charging tRNAs with their cognate amino acids in protein translation and KARS, one of the two bifunctional tRNA synthetases, is essential for protein synthesis in both the cytosol and mitochondria (86). Further studies have demonstrated that the interaction with mutant SOD1 leads to an increased misfolding and aggregation of KARS prior to its import into mitochondria, resulting in decreased mitochondrial protein synthesis and abnormal morphology and cell toxicity (85).

Mitochondria sustain damage with aging. One of the early steps in mitochondrial quality control (MQC) is the translocation of Parkin, an E3 ubiquitin-protein ligase which is linked to Parkinson's disease, from the cytosol to the mitochondria, leading to the recruitment of autophagy receptors to the mitochondrial surface and causing changes in mitochondrial distribution (87). To be specific, Parkin translocation promotes the K63-linked polyubiquitination of mitochondrial substrates (such as Mfn2 and Miro1) and recruits the ubiquitin- and LC3-binding autophagy receptor, p62/SQSTM1, to the mitochondria (87). Interestingly, in spinal cord motor neurons of SOD1-G93A mice, a similar situation was reported (88). Therefore, in the cytosol, Parkin has been shown to mediate K63-linked polyubiquitination of SOD1 mutants in cooperation with the UbcH13/Uev1a E2 enzyme, resulting in the recruitment of p62/SQSTM1 (via its ubiquitin-association domain) to form aggregates (89). It is conceivable that SOD1 mutant may translocate to the mitochondria together with Parkin and/or p62/SQSTM1, which have been shown to bind directly to each other (90). As mentioned above, the SOD1 mutant can accumulate on the mitochondrial surface by binding directly to Bcl-2 and/or VDAC1, but it is not clear whether binding to Bcl-2 and/or VDAC1 is dependent on the ubiquitination state of the SOD1 mutant or not. Moreover, it is unclear whether the association with the SOD1 mutant results in a dysfunctional p62/SQSTM1. It has been shown that p62/SQSTM1 deficiency is associated with inhibited complex I mitochondrial respiration due to a lack of NADH for the electron transport chain. This deficiency is also associated with increased levels of NADPH reflecting a higher activation of the pentose phosphatase pathway resulting in higher cytosolic reduced glutathione levels and higher cytosolic ROS production (91).

Inside mitochondria, wild-type SOD1 has been suggested to inhibit mitochondrial metabolism at the respiratory complex I within the intermembrane space (IMS) in a K122 dependent manner. Suppressing or boosting respiration levels toggled SOD1 in or out of the mitochondria, respectively (92). Mutant SOD1 has been reported to accumulate in the IMS and in the matrix of neural tissue and cause mitochondrial dysfunction without any known direct interaction partners (12, 93, 94). It has been reported that in vacuolated mitochondria mutant SOD1 colocalizes with cytochrome c; however, whether there is a physical interaction is not known (95). Interestingly, mitochondria-associated membrane (MAM), an interface between the mitochondria and the ER, has been identified to associate with mutant SOD1, leading to a loss of interaction between the sigma 1 receptor (Sig1R) and the IP3 receptor 3 (IP3R3), dissociation of the mitochondria and the ER, and dysregulation of Ca^2+^ homeostasis etc. However, evidence shows that SOD1 neither binds to Sig1R nor IP3R3 at the MAM (96). Since ER stress sensors such as PERK and BiP, are enriched at the MAM (97), mutant SOD1 proteins accumulated at the MAM may trigger an ER stress response.

### SOD1 Mutant Interaction Partners Related to ER Stress

It has been shown that mutant SOD1 accumulates inside the ER of transgenic mice spinal cords where it forms insoluble high molecular weight species and interacts with the ER chaperone binding immunoglobulin protein (BiP/HSP70) and activates the adaptive signal transduction pathways, including the unfolded protein response (UPR) (98). However, neither genetic inhibition of the UPR via ablation of doublestranded RNA-activated protein kinase-like ER kinase (PERK), nor genetic UPR enhancement via ablation of growth arrest and DNA damage-inducible protein 34 (GADD34/PPP1R15A), is beneficial for mutant SOD1-induced motor neuron disease, suggesting that the UPR-PERK pathway is not a likely target for therapeutic intervention in ALS (99). Interestingly as reported, SOD1 mutants commonly interact with the ER-resident membrane protein Derlin-1, triggering motoneuron death and contributing to the pathogenesis of ALS (100, 101). Derlin-1 is a component of the ER-associated degradation (ERAD) machinery, is involved in the translocation of misfolded proteins from mammalian ER into the cytosol (102) and/or rerouting of the misfolded substrate to the proteasome through interactions with the signal recognition particle (SRP) (103). It has been shown that an interaction between SOD1 mutants and Derlin-1 triggers ER stress through dysfunction of ERAD, leading to ASK1 activation and motor neuron death, thereby controlling the disease progression of ALS (100). However, this mechanism has not been described earlier in pathology and it was found that mutant SOD1 interacts with the coatomer coat protein II (COPII) and causes impaired ER-Golgi transport before induction of ER stress (104). Translocon-associated protein delta (TRAPD/SSR4), another ER membrane resident protein responsible for calcium binding, was also proven to specifically interact with SOD1 mutants (84), which may contribute to the dysregulation of Ca^2+^ homeostasis together with the abnormal IP3R3 signaling triggered by SOD1 mutant.

### SOD1 Mutant Interaction Partners Related to Aberrant RNA Processing

Vascular endothelial growth factor (VEGF), originally described as a key angiogenic factor, is also a neuroprotective growth factor essential for the maintenance of motor neurons (105). Decreased VEGF gene expression in mice results in a phenotype similar to that seen in patients with ALS, thus linking loss of VEGF to the pathogenesis of motor neuron degeneration and PI3K/Akt signaling (106). It has been shown that SOD1 mutants bind, by competing with the ubiquitous RNA-binding and stabilizing protein (RBP) HuR, directly to the VEGF 3′-untranslated region (UTR) with a predilection for adenine/uridine-rich elements (ARE) (107, 108), leading to dysregulation of VEGF mRNA stability and downregulation of VEGF (109). Wild-type SOD1 also binds to the ARE of VEGF, but very weakly, and the SOD1 N terminal amino acids 32-49 are proven to be responsible for this binding affinity (107, 108).

Neurofilaments play a critical role in the maintenance of neuronal caliber. NF-L is the most abundant of the three neurofilament proteins (NF-L, M, H) and like the other non-epithelial intermediate filament proteins, it can form homopolymeric 10 nm filaments. It not only provides structure and mechanoresistance but also provides a scaffolding for the organization of the nucleus and organelles such as mitochondria and ER. Importantly, failure to properly assemble into a filamentous network or disruption of expression and metabolism of neurofilament proteins is characteristic of certain neurological syndromes including ALS, Charcot-Marie-Tooth sensorimotor neuropathies, Giant Axonal Neuropathy, Alzheimer's and Parkinson's disease (110, 111). With regards to ALS, it was found that NF-L mRNA levels are selectively suppressed and that mutant SOD1, TDP-43, and 14-3-3 can each function as NF-L mRNA 3′-UTR binding proteins that directly affect the stability of the NF-L transcripts. Furthermore, NF-L mRNA is preferentially sequestered to both stress granules (TIA-1 immunoreactive) and processing bodies (XRN-1 immunoreactive) in ALS (112). Interestingly, there is a report showing that SOD1 mutants can physically interact with TDP-43 in an RNA-independent manner, and the region responsible for this interaction within SOD1 is the dimer interface, namely the N- and C-terminal regions (113). Considering the features of NF-L mRNA binding, these results suggest that the binding region where SOD1 mutant binds to the NF-L mRNA 3′-UTR is not the dimer interface since it is occupied by TDP-43. Recently, another report showed that SOD1 mutants have no physical interaction with TDP-43 (114), thus SOD1 mutants and TDP-43 may each function as NF-L mRNA 3′-UTR binding proteins as mentioned above, and they might competitively or synergistically affect the stability of the NF-L transcripts. It is known that TDP-43 is important for RNA homeostasis regulation in ALS, but the exact mechanisms are unknown. On one side, overexpression of TDP-43 is claimed to lead to RNA destabilization and reduction in mitochondrial components (115), on the other side, loss of TDP-43 is claimed to decrease the levels of Atg7 and HDAC6 mRNAs causing impaired autophagy (116, 117), indicating that TDP-43 may play either an RNA stabilizing or destabilizing role dependent on the scenario, such as in the presence or absence of an SOD1 mutant.

The 14-3-3 proteins are a family of chaperones comprising ~1% of the whole brain proteins (118). As discussed above, 14-3-3 is one of the NF-L mRNA 3′-UTR binding partners, and has also been found to partially localize in the Lewy body-like hyaline inclusions (LBHI) of spinal cords with fALS and in mutant SOD1 transgenic mice, suggesting that 14-3-3 proteins are involved in the formation of both SOD1 mutants containing inclusions and stress granules (119). In N2A neuroblastoma, 14-3-3 was found to co-localize with SOD1 mutant aggregates in the JUNQ compartment (120). However, it is not clear whether SOD1 mutants bind directly to 14-3-3, and whether the formation of LBHI and stress particles depends on the interaction between mutant SOD1 and 14-3-3. Interestingly, SOD1 mutants were found to directly interact with the GTPase-activating protein SH3 domain-binding protein 1 (G3BP1), a stress granule marker which binds to RNA, in an RNA-independent manner. It has been claimed that this interaction consequently delays the formation of G3BP1- and TIA1- positive stress granules, suggesting that pathogenic SOD1 mutations can potentially alter RNA metabolism in ALS besides their effects on VEGF and NF-L mRNAs (114). Interestingly, cyclic GMP-AMP synthase (cGAS), a key sensor responsible for cytosolic DNA detection and autoimmune response, was also revealed to interact with G3BP1 for its efficient priming and activation (121). Whether mutant SOD1 competes for G3BP1 binding with cGAS and affects priming/activation of the latter is not clear, but needs to be clarified.

### SOD1 Mutant Interaction Partners Related to Protein Degradation

Eukaryotic cells have two major protein degradation pathways: the ubiquitin-proteasomal system (UPS) and the autophagy-lysosomal system. Proteasome is a multimeric ATP-dependent protease complex composed of a core 20S protease capped with two 19S regulatory subunits that selectively degrade unnecessary or damaged ubiquitinated proteins by proteolysis (a chemical reaction that breaks peptide bonds) (122–124). Whereas, lysosome is involved in autophagy which allows the orderly degradation and recycling of aggregated proteins and cellular components (125, 126). Three forms of autophagy are commonly described: macroautophagy, microautophagy, and chaperone-mediated autophagy (CMA) (127). It has been reported that the ALS-related SOD1 mutant binds directly to the S6 (Rpt3/PSMC4) and S6′(Rpt5/PSMC3) subunits of the 19S regulatory complex of the proteasome and interferes with their normal functions. Since both S6 and S6′ subunits belong to the AAA ATPase superfamily, it has been suggested that the association between these ATPase subunits and the mutant SOD1 proteins impairs the ATPase-induced activation of the 20S catalytic core and causes proteasomal dysfunction and degradation stalling (128). Moreover, the SOD1 mutant interacts physically with the co-chaperone CHIP (carboxyl terminus of Hsc70 interacting protein) (129, 130) and with the ubiquitin-binding proteins Bag1 and VCP (valosin-containing protein; p97) (128). Interestingly, CHIP and VCP compete with each other for the binding to SOD1 mutant, indicating that the chaperone complex (CHIP/Hsp70) and the proteolytic machinery (VCP/26S proteasome) compete for the common substrate SOD1 mutant (128). As CHIP provides a link between the chaperones and UPS and probably regulates the balance between protein refolding and degradation, it is unclear whether its association with mutant SOD1 will hinder its function as the intermediary, thereby aggravating UPS dysfunction and causing protein homeostasis (proteostasis) deficiency. However, the level of CHIP has been observed to be declined in SOD1 H46R/H48Q transgenic mice (67).

Besides the co-chaperone CHIP, SOD1 mutants can also directly interact with chaperones (heat shock proteins; Hsps) and lead to the formation of sedimentable aggregates, hence it has been even proposed that the binding of Hsps to SOD1 mutants makes Hsps unavailable or dysfunctional for their antiapoptotic effects and ultimately leads to motor neuron death (131, 132). However, in another report, although most of the proteins bound to an immobilized SOD1 mutant (apo G93A) in spinal cord extracts were found to be chaperones, by far the most abundant was Hsc70, but only about 1% of the total Hsc70 appeared to be associated with the misfolded SOD1. Therefore, this result refutes the view that chaperone depletion is involved in ALS pathogenesis in transgenic models and in humans carrying SOD1 mutations (133). Clearly, the binding of SOD1 mutants to Hsc70 seems unlikely to cause its depletion. Most probably the coupling of SOD1 mutants with Hsc70 is necessary for promoting the degradation of the SOD1 mutants by facilitating the translocation of the substrates from the 19S regulatory particles (for recognition and unfolding) to the 20S core particles (for degradation) via cooperation with the co-chaperone CHIP. In fact, CHIP was reported to specifically reduce the association between SOD1 mutants and the S6/S6′ATPase subunits of 26S proteasomes, which may account for the co-chaperone‘s ability to promote degradation of the aggregation-prone SOD1 mutants (128).

In the list of binding partners of SOD1 mutants related to protein degeneration, there is another member, NEDL-1(NEDD-4-like ubiquitin-protein ligase-1), a novel ubiquitin-protein isopeptide ligase, which has been shown to be associated with translocon-associated protein-delta. Interestingly, SOD1 mutants also physically interact with Disheveled-1, the substrate of NEDL-1 and one of the key transducers in the Wnt signaling pathway, in the presence of NEDL1 and to cause its dysfunction, indicating that SOD1 mutants can function as organizers for the formation of complexes that promote degradation of the SOD1 mutants by the UPS and also affect the functions of components such as those involved in Wnt signaling, ER stress and the proapoptotic activity of p53 (134, 135).

As is known, autophagy is another important branch of protein degradation. In this respect, SOD1 mutants have been reported to interact with Parkin and p62/SQSTM1 to cause autophagy of misfolded proteins in motor neurons (88, 90, 136). As reported, Parkin is responsible for mediating ubiquitination of SOD1 mutants in cooperation with the UbcH13/Uev1a E2 enzyme and the recruitment of p62/SQSTM1 for aggregate production, whereas p62/SQSTM1 allows the linking of mutant SOD1 to LC3, thus indicating that p62/SQSTM1 is involved in both aggresome and autophagosome formation (136). During proteasome impairment, p62/SQSTM1 is demonstrated to activate the autophagy-lysosomal system by promoting sequestration of misfolded and aggregated SOD1 proteins to form perinuclear aggresomes and facilitating aggresome clearance by autophagy. In aggresome formation, the ubiquitin-association (UBA) domain of p62/SQSTM1 is shown to be necessary (89), whereas in autophagosome formation, three alternative domains of p62/SQSTM1 are involved: the SMIR region (AA 178-224, responsible for the interaction with mutant SOD1), the PB1 domain (AA 1-104, responsible for oligomerization of p62/SQSTM1) and a domain responsible for LC3 binding (90). Age-dependent neurodegenerative diseases like ALS are associated with a decline in protein quality control systems including autophagy. Considering the underlying mechanism of ALS, there is always a recurring theme of protein misfolding as in other neurodegenerative diseases (137). Thus, it is conceivable that in SOD1 mutant-caused ALS, the overall decline of autophagic flux may be induced by the association of SOD1 mutants with p62/SQSTM1, although the association of the two can promote autophagy to remove misfolded SOD1 mutants to some extent in the early disease stages. This is consistent with the report that motor neurons, initially able to maintain the misfolded SOD1 mutant in a soluble state, become progressively unable to do so (138).

### SOD1 Mutant Interaction Partners Related to Axonal Transport Defects

Axonal transport is an essential process in neurons, and is responsible for the movement of mitochondria, lipids, synaptic vesicles, proteins, and other cellular organelles to and from a neuron's cell body (139). Traditionally, two main classes of axonal transport have been distinguished based on the overall speed of the movement, namely fast, and slow axonal transport. Both of them are mediated by the same molecular motors that move cargos along microtubes and neurofilaments in both directions between the axonal terminals and the cell body (140). The outgoing anterograde transport from the cell body to the axon terminal and ingoing retrograde transport the other way round are driven by kinesin and dynein, respectively (141, 142).

Results from vesicle motility assays in an isolated squid axoplasm indicated that the fALS-linked-SOD1 H46R mutant selectively inhibits conventional kinesin-based fast axonal transport (FAT) in the anterograde direction, whereas the WT SOD1 protein does not affect FAT in either the anterograde or retrograde direction. Moreover, biochemical and pharmacological experiments have further shown that SOD1 mutant inhibits FAT through a mechanism involving specific activation of p38 MAPK. However, the direct interaction between mutant SOD1 and p38 MAPK is not shown (62). Another study demonstrated that dynein-based retrograde transport is disrupted by the aberrant interaction between SOD1 mutants (SOD1 A4V, G85R, and G93A) and the dynein complex. Moreover, quantitative results have shown that the amount of bound SOD1 mutant increases during disease progression prior to the onset of the symptoms in both motor neuron cell lines and transgenic mice (143), suggesting that the dynein-mediated retrograde transport dysfunction may play an important role in the SOD1 mutant-mediated fALS etiology.

Microtubules are polymers made up of tubulin which itself is a heterodimer of α-tubulin and ß-tubulin. Tubulin is one of the major SOD1 mutant-interacting proteins. The interaction between tubulin and SOD1 mutants was detected in the spinal cords of mutant G93A SOD1 transgenic mice before the onset of any symptoms. Interestingly, the N-terminus (AA 1-23) and C terminus (AA 116-153) of SOD1, which are the regions responsible for dimerization within SOD1 (they usually form the dimer interface), have been shown to interact directly with tubulin (144), indicating that the tubulin- bound SOD1 mutant form is most likely a reoriented dimer or a monomer with an exposed interface, and the monomer of the misfolded SOD1 has been proven to be more adherent to inadequate protein-protein interactions (145). Interestingly, histone deacetylase 6 (HDAC6), a tubulin deacetylase, also selectively interacts with SOD1 mutants (146), suggesting that mutant SOD1 can modulate HDAC6 activity and increase tubulin acetylation, which, in turn, facilitates microtubule-dependent SOD1 aggregation and deficient axonal cargo transportation.

Additionally, SOD1 mutants selectively interact with neurofilaments (133, 147). The neurofilament type seems to directly control axonal diameter, which in turn controls how fast electrical signals and material transport travel down the axon (141). In contrast to dynein, tubulin and neurofilament type, SOD1 mutants do not interact with anterograde transport motors of the kinesin-1 family (KIF5A, B, and C). It has been shown that dynein-mediated retrograde transport was slower in SOD1 G93A than in WT mice at an early pre-symptomatic stage. While no decrease in KIF5A-mediated anterograde transport was detected, the anterograde transport of dynein heavy chain as cargo was observed in pre-symptomatic G93A mice, implying that SOD1 mutants might only interact with and interfere with some kinesin members which in turn could result in the impairment of a selective subset of cargos. Although it remains to be further investigated how SOD1 mutants affect different axonal transport motor proteins and various cargos, it is evident that SOD1 mutants can induce defects in axonal transport, which subsequently contribute to the propagation of toxic effects and ultimately motor neuron death in ALS (148).

### SOD1 Mutant Interaction Partners Related to Other Cellular Dysfunction

Shortly after the discovery of SOD1 as the first gene to be linked to ALS, the reduction of the presynaptic marker synaptophysin in the ventral horn of ALS subjects and the depletion of synaptic vesicles in the neuromuscular junction have been shown to be the earliest pathological events in SOD1 G93A mice (149, 150). To investigate the underlying mechanism of these findings, the adaptor-associated kinase 1 (AAK1), a regulatory protein in the clathrin-coated vesicle endocytic pathway, has been reported to selectively colocalize with SOD1 mutants but not with wild-type SOD1. However, the authors have not provided evidence showing the direct protein-protein interaction between AAK1 and mutant SOD1 proteins. It is suggested that the two may exist in the same hyaline aggregate via intermediate molecules, suggesting that SOD1 mutants may exert their toxicity via sequestration of AAK1, thus affecting the endosomal and synaptic vesicle recycling pathways. More experiments are warrant to determine whether or not SOD1 mutants directly interact with AAK1. It has been reported that the SOD1 G93A mutation results in abnormal mitochondrial morphology and a reduced size of the synaptic vesicle pool in the presynaptic terminals (151). Whether and to which extent the two events are correlated to each other is not clear. Furthermore, SOD1 mutants have also been found to interact with chromogranins, components of neurosecretory vesicles, in immune-isolated trans-Golgi network and in microsome preparations, suggesting that SOD1 mutants can be secreted. It has been observed that extracellular SOD1 mutants can trigger microgliosis and neuronal death (152). Interestingly, extracellular fragments of Glycoprotein non-metastatic melanoma protein B (GPNMB) secreted from activated astrocytes have been found to attenuate the neurotoxicity of SOD1 G93A (153). GPNMB is a type I transmembrane protein and has been identified as another SOD1 mutant interaction partner. It is especially expressed in motor neuron and astrocytes, and in NSC-34 cells, its glycosylation is inhibited by direct interaction with SOD1 G93A, resulting in an increase in motor neuron vulnerability (153).

## Conclusion

A large number of studies have shown that SOD1 has many naturally occurring mutants, and the molecular morphological changes caused by various mutations are also different. Except for zinc and copper ions, the molecules that interact directly and/or indirectly with SOD1 mutants are also widespread. It is clear that many mutations can make SOD1 become destabilized and toxic to cells, but the toxicity mechanism is obviously very complex and diverse. However, some commonalities have been found in the structures of the various mutants, that is, the regions which lead to unusual interactions with other molecules are concentrated in the dimeric interface area and the electrostatic loops. Nevertheless, the roles they play in cytotoxicity are not fully understood. It is also not clear which parts of the mutant molecules are the main building blocks and which parts play dominant roles in the cell death and disease process.

Furthermore, although most SOD1 mutants have been shown to exist as dimers in solution according to registered structural reports, it is also possible that the same SOD1 mutants present distinct forms at different stages in the maturation process, and that these distinct forms may present distinct epitopes and bind with different molecules. Therefore, it will be useful to clarify which SOD1 mutant forms can bind with which molecule at which stage. However, due to the complexity of the molecular morphology in the soluble state, the diversity of the mutations and the specificity of the tissues, it is still difficult to fully achieve this goal. Recently, soluble SOD1 mutant forms have been suggested to be more toxic than aggregate forms, and all the interaction molecules listed in this review have been shown to interact with soluble forms of SOD1 mutants, thus further identification and narrow-down of the binding epitopes (segments) of the SOD1 mutants with other molecules should be meaningful for unraveling the origins of disease pathology. If we can accurately map the domains of the SOD1 mutant molecules involved in toxicity, this will provide a solid basis for drug design and interference.

Moreover, SOD1 mutants have been shown to be easily misfolded and become adherent for binding with themselves and with other molecules to form aggregates. Interestingly, although protein aggregation occurs in all cells, SOD1 mutants have been invariably characterized to be selectively toxic to motor neurons. Cells have evolved an elaborate quality control system to ensure the correct folding of proteins and avoid the abnormal binding of protein intermediates with other molecules. How the quality control system for misfolded proteins in a motor neuron is disarmed and leads to selective toxicity in this particular context needs to be extensively studied. Compared with other cells, one of the prominent morphological features of motor neurons is the long axon. Due to their extended length, material transport is problematic. Therefore, the toxicity of the SOD1 mutants to mitochondria, the endoplasmic reticulum and other cell organs or molecules may have serious consequences. Not only is the removal of defective components at the distal end of the axon problematic, but also the removal of obstacles is difficult due to the distance from the cell body. Therefore, while we emphasize the discovery of the toxic epitopes of the SOD1 mutants, we need to deeply examine the transport and compartmentalization of SOD1 mutants in motorvad neurons, and identify how and where these proteins contact one another to initiate the toxicity in motor neurons and its accumulation process, in order to provide targeted treatment for specific diseases.

## Author Contributions

JH designed the structure and wrote the first draft of the manuscript. The manuscript was further revised by JH along with input from ZZ. All authors contributed in revising the initial draft, as well as reviewing, preparing and approving the final draft of the manuscript for submission.

### Conflict of Interest Statement

The authors declare that the research was conducted in the absence of any commercial or financial relationships that could be construed as a potential conflict of interest.
